# Efficacy of a Multicenter Hospital Network's Approach to Enacting Blood Culture Stewardship During a Global Shortage

**DOI:** 10.1093/ofid/ofaf294

**Published:** 2025-05-16

**Authors:** Matthew J Shelly, Anna Kunz, Jaimie Mittal, Douglas S Corwin, Christopher Chapman, Peter T Ender

**Affiliations:** Department of Medical Education, Temple/St Luke's School of Medicine, Bethlehem, Pennsylvania, USA; Department of Medical Education, Temple/St Luke's School of Medicine, Bethlehem, Pennsylvania, USA; Section of Infectious Diseases, Department of Medicine, St Luke's University Health Network, Bethlehem, Pennsylvania, USA; Section of Pulmonary and Critical Care, Department of Medicine, St Luke's University Health Network, Bethlehem, Pennsylvania, USA; Department of Pathology and Laboratory Medicine, St Luke's University Health Network, Bethlehem, Pennsylvania, USA; Section of Infectious Diseases, Department of Medicine, St Luke's University Health Network, Bethlehem, Pennsylvania, USA

**Keywords:** blood culture bottles, blood cultures, diagnostic stewardship, sepsis, supply shortage

## Abstract

The recent global blood culture bottle shortage forced hospitals to implement new diagnostic stewardship programs. In this brief report, we describe our approach as a multicenter hospital network through a system-wide memorandum and the implementation of a best practice advisory, which decreased the use of blood cultures without negatively affecting the sepsis-related mortality rate.

Bloodstream infections (BSIs) represent a common infectious cause of disease and death, with an average of 2 million cases and 250 000 deaths occurring annually in North America and Europe [1]. Blood cultures remain the definitive diagnostic for identifying BSI and are a necessary component of the sepsis pathway, as defined by the Centers for Medicare & Medicaid Services Severe Sepsis and Septic Shock Early Management Bundle (SEP-1) measure [2].

In clinical scenarios beyond the predetermined SEP-1 measure, the use of blood cultures varies greatly among providers. Expert guidelines exist to aid clinicians in determining optimal usage of blood cultures [3]. Despite this guidance, the literature suggests that fever and leukocytosis are often the sole clinical justification for obtaining blood cultures, resulting in unnecessary medical interventions and financial burden [4]. To address this potential overuse, institutions often develop clinical diagnostic algorithms or best practice advisories (BPAs) [5]. The effectiveness of these interventions varies, with some having low to moderate sensitivity and suboptimal positive predictive value for detecting BSI [5]. BPAs have successfully reduced blood cultures orders by >33% without sacrificing Centers for Medicare & Medicaid Services compliance; however, these studies lack secondary outcomes such as sepsis-related deaths and occurred outside of a global shortage [6].

In June 2024, Becton Dickson and Company (BD) notified customers of a global shortage of BACTEC blood culture bottles [7, 8]. As our institution's only blood culture supplier at the time, this shortage forced a reevaluation of our blood culture ordering practices. This brief report illustrates how our hospital network addressed this shortage through a system-wide memorandum and implementation of BPA within our electronic health record (EHR), while assessing the effect on patient outcomes.

## METHODS

### Setting and Study Population

St Luke's University Health Network includes 12 acute care hospital campuses with level I through level IV trauma centers spread across Pennsylvania and New Jersey. The data presented in this study combine emergency department encounters, hospital observations, and hospital admissions from patients (>18 years of age) across the 12 campuses regardless of presenting diagnosis, immunocompetency, or severity of illness. Pediatric patients (<18 years of age) were excluded.

### Interventions

On 23 June 2024, a network-wide memorandum was sent via electronic mail to all medical and nursing staff detailing the current blood culture bottle shortage. Within this memorandum were reminders regarding proper collection protocol and a list of inappropriate justifications for blood cultures visible in Supplementary Table 1 (Supplementary Table 1). On 3 July 2024, a BPA was added to the EHR blood culture order set, which also contained the information within Supplementary Table 1. This BPA was a full-screen pop-up message which required acknowledgement of the information and provided clinicians the opportunity to remove the blood culture order.

### Data Sources

**Figure 1.**
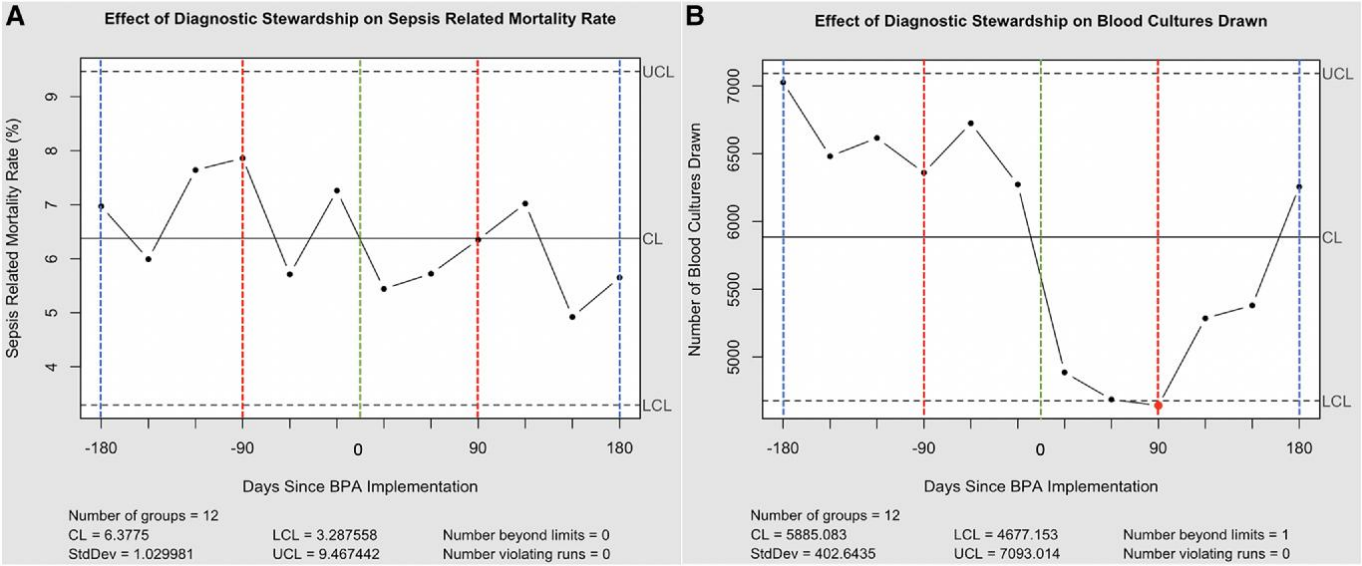
Sepsis mortality rates and blood culture orders across St Luke's University Health Network. Individual–Moving Range charts of sepsis mortality rates (*A*) and blood culture orders (*B*), before and after implementation of the best practice advisory, were created using the qcc package within R software (version 2024.09.1). The data point at 90 days in *B* represents a month “beyond limits,” with further explanation available elsewhere [11]. The center line (CL; mean), upper confidence level (UCL)/lower confidence level (LCL) (±3 SDs), and SD were computed automatically using the qcc package.

Patients with a sepsis diagnosis were identified based on *International Classification of Disease, Tenth Revision* (*ICD-10*) diagnostic codes. Observed deaths were recorded based on discharge status, and the monthly sepsis mortality rates were calculated (Figure 1*A*). The expected mortality rate was calculated with a logistic regression model which examined patient characteristics and conditions at time of admission [9]. The hospital network's blood culture data were provided by the microbiology department. The total number of blood cultures obtained, the number of individual patients with blood cultures, the number of positive blood cultures, and the number of contaminated blood cultures per month were reviewed.

### Study Approval

The design of the study was approved by the St Luke’s University Health Network Institutional Review Board (IRB), and IRB exemption was obtained for this deidentified, population-based research study.

### Statistical Analysis

The sepsis mortality rates and the numbers of patients with blood cultures during the 90 and 180 days before and after 3 July 2024 were compared using a χ^2^ test with an α level of .05. Individual–Moving Range (I-MR) charts for both sepsis-associated deaths and blood cultures obtained against time were generated using the qcc package in R software, version 2024.09.1 [10, 11].

## RESULTS

### Overview of Blood Culture Testing and Patients With a Diagnosis of Sepsis

From 4 April 2024 to 1 October 2024 (from 90 days before to 90 days after the BPA) there were 199 124 total patient encounters, during which 33 125 blood cultures were obtained, with 2981 (8.99%) identified pathogens and an average contamination rate of 2.14%. Sepsis was diagnosed in 4718 patients, with a mortality rate of 6.08%. From 5 January to 30 December 2024 (from 180 days before to 180 days after the BPA) there were 398 146 total patient encounters, during which 70 621 blood cultures were obtained, with 6168 (8.73%) identified pathogens and an average contamination rate of 1.95%. Sepsis was diagnosed in 9560 patients, with a mortality rate of 6.43%.

### Blood Culture and Sepsis Data Before and After Education/BPA

The effects of the education initiative and EHR BPA implementation are summarized in Table 1. The 90-day pre/post-BPA analysis revealed a significant decrease in both the total number of blood cultures and the number of patients with blood cultures after the BPA was implemented (7566 [7.60%] vs 4961 [4.98%] patients; χ^2^[1; n = 199 124] = 572.05; *P* < .001). Despite the decrease in both the total number of blood cultures and the number of patients with blood cultures, there was no significant difference in sepsis-related mortality rate between the 2 time periods (6.81% vs 5.83% (χ^2^[1; n = 4482] = 0.84; *P* = .36).

**Table 1.** Blood Culture and Sepsis-Related Mortality Summary Data

Variable	90-d Analysis^a^				180-d Analysis^a^			
	Before BPA	After BPA	Change, %	*P* Value	Before BPA	After BPA	Change, %	*P* Value
Total patient population, no.	99 496	99 628	0.13	…	197 626	200 520	1.46	…
Patients with blood cultures, no.	7566	4961	−34.43	.001	14 203	11 831	−16.70	.001
Total blood cultures, no.	19 358	14 217	−26.56	…	39 481	31 140	−21.13	…
Blood culture contamination rate, %	1.77	2.5	41.26	…	1.8	2.1	16.67	…
Patients with diagnosed sepsis, no.	2322	2160	−6.98	…	4888	4672	−4.42	…
Sepsis-related mortality rate, %								
Observed	6.81	5.83	−14.39	.36	6.83	5.86	−14.20	.09
Expected	8.45	9.01	6.63	…	8.98	8.47	−5.68	…

Abbreviation: BPA, best practice advisory.

^a^Data for the 90- and 180-day analyses before and after the BPA initiative on 3 July 2024.

The 180-day pre/post-BPA analysis also revealed a significant decrease in the total number of blood cultures and the number of patients with blood cultures after the BPA was implemented (14 203 patients [7.19%] vs 11 831 [5.90%]; χ^2^[1; n = 398 146] = 269.18; *P* < .001). Despite the decrease in both the total number of blood cultures and the number of patients with blood cultures obtained, there was no significant difference in sepsis-related mortality rate between the 2 longer time periods (6.83% vs 5.86% (χ^2^[1; n = 9560] = 2.79; *P* = .09). In the final month of the analysis, the total number of blood cultures returned to the baseline level seen before the BPA (Figure 1*B*).

## DISCUSSION

In the setting of a global shortage of blood culture bottles, use of a network-wide memorandum in blood culture stewardship and a BPA in the EHR significantly reduced the quantity of total blood cultures and the number of patients with blood cultures across 90 and 180 days, respectively. Despite the decrease in the use of blood cultures, the sepsis-related mortality rate did not increase significantly across either time period. This outcome is comparable to that in previous studies that implemented similar clinical decision supports and decreased the number of blood cultures by 29.5% [12] and 33.5% [6].

Although initially successful, the effectiveness of these interventions diminished as the number of blood cultures in the sixth month after BPA (December) increased 25% compared with the third month after BPA (September), returning the number to pre-BPA levels (Figure 1*B*). This 25% increase in blood cultures is beyond typical seasonal variation [13] and 11% greater than the same time period in 2023 despite controlling for the absolute number of patients (data not shown). This suggests that the favorable short-term effect of education on clinician ordering behavior may decline over time, possibly due to BPA fatigue, a lack of repetitive reeducation, or a belief among ordering clinicians that the critical shortage had resolved. A similar loss of education efficacy over time after halting monthly blood culture stewardship has been described elsewhere[14].

The return to prestudy blood culture levels after 6 months demonstrates the need for a recurring, multifaceted approach to diagnostic stewardship. Additional interventions that may help sustain lower blood culture levels include quality-focused algorithms involving nursing staff, continuous clinician reeducation, and EHR restrictions on certain blood culture practices. By engaging nursing staff, one can ensure sample collection quality through adequate blood volume, sterile technique, and the type of vascular access best suited for blood culture [15]. Nursing-driven quality assurance has shown to decrease contamination rates and follow-up culture collection by nearly 2.0% [16]. Continuous reeducation clinicians may also be effective, especially those in the emergency department, where some studies suggest that 20%–25% of blood cultures are taken [17, 18]. EHR restrictions on repeated blood cultures in situations with minimal impact on patient care, such as gram-negative BSIs, may also lead to decreased blood culture orders [19]. However, electronic interventions that affect ordering autonomy may not be received well by clinicians and could affect quality outcomes. Despite this concern, a recent systematic review of this approach concluded that clinicians are generally tolerable of EHR restrictions, if they are involved in the guideline creation process [20].

This study is not without limitations. The quasi-experimental design comparing postintervention with historical outcomes may mask potential confounding factors better controlled for within a randomized comparison. Clinical outcomes beyond sepsis-related deaths were not studied and may have been affected. Given the 6-month time constraint on the data, longer-term outcomes were unquantifiable. Despite these limitations, the short-term change in blood culture ordering provides compelling support for this type of intervention.

In conclusion, the current study highlights a successful approach to decreasing the use of blood cultures in the setting of a global shortage, through a network-wide memorandum and implementation of a provider-focused BPA. This approach resulted in no negative impact on the sepsis-related mortality rate. Other approaches to blood culture stewardship have been described, but this analysis provides longer follow-up data of 180 days and includes clinical outcomes. Moving forward, our hospital network will consider other diagnostic stewardship strategies to maintain these successful outcomes over the long-term.

## Supplementary Material

ofaf294_Supplementary_Data

## Supplementary Data

Supplementary materials are available at *Open Forum Infectious Diseases* online. Consisting of data provided by the authors to benefit the reader, the posted materials are not copyedited and are the sole responsibility of the authors, so questions or comments should be addressed to the corresponding author.
