# Supplementary material for: Efficacy of a Multicenter Hospital Network's Approach to Enacting Blood Culture Stewardship During a Global Shortage
Source: Open Forum Infect Dis. 2025 May 16;12(6):ofaf294. doi: 10.1093/ofid/ofaf294 (PMC12125671; doi:10.1093/ofid/ofaf294)
Supplement: ofaf294_Supplementary_Data [file ofaf294_supplementary_data.docx]

**Supplementary Material:**

**Supplementary Table 1 (Table S1).** Electronic Health Record Best Practice Advisory (BPA)

*At present, there is a national manufacturing shortage of blood culture bottles. To facilitate stewardship of blood culture bottles and maintain adequate stock, the infectious disease service and microbiology laboratory offers the following guidance:*

| The following use of blood cultures are usually unnecessary or inappropriate: |
| --- |
| 1. Repeat blood culture for uncomplicated Gram-negative Bacteremia |
| 1. Repeat blood culture when only 1 of 2 blood cultures are positive for Staphylococcal species, other than Staphylococcus aureus or lugdunensis, in a patient with no intravascular device in place |
| 1. Repeat blood cultures for patients with negative blood cultures within the past 5 days |
| 1. Repeat blood cultures done prior to 48 hours of effective treatment when confirming clearance of a Gram-positive bacteremia |
| 1. Blood cultures done in a patient with a localized infection such as soft tissue infection or UTI without clinical sepsis |
| 1. Blood cultures drawn through a vascular catheter, with the exception of those done with hemodialysis |

**Supplementary Table 1.** The BPA above appears when a clinician attempts to order a set of blood cultures for a patient. After reviewing the list, the clinician can opt to keep or cancel the order as deemed appropriate. These guidelines are based on the current standard of practice and are comparable to other diagnostic stewardship campaigns within the literature.
